# Thin Layer-Protected Gold Nanoparticles for Targeted Multimodal Imaging with Photoacoustic and CT

**DOI:** 10.3390/ph14111075

**Published:** 2021-10-25

**Authors:** Jing Chen, Van Phuc Nguyen, Sangeeta Jaiswal, Xiaoyu Kang, Miki Lee, Yannis M. Paulus, Thomas D. Wang

**Affiliations:** 1 Division of Gastroenterology, Department of Internal Medicine, University of Michigan, Ann Arbor, MI 48109, USA; chenjingsimm@gmail.com (J.C.); jaiswals@umich.edu (S.J.); kangx@umich.edu (X.K.); leemiki@umich.edu (M.L.); 2 Kellogg Eye Center, Department of Ophthalmology and Visual Sciences, University of Michigan, Ann Arbor, MI 48109, USA; vanphucn@umich.edu (V.P.N.); ypaulus@umich.edu (Y.M.P.); 3 Department of Biomedical Engineering, University of Michigan, Ann Arbor, MI 48109, USA; 4 Department of Mechanical Engineering, University of Michigan, Ann Arbor, MI 48109, USA

**Keywords:** nanoparticle, multimodal imaging, photoacoustic, heterobivalent peptide

## Abstract

The large size of nanoparticles prevents rapid extravasation from blood vessels and diffusion into tumors. Multimodal imaging uses the physical properties of one modality to validate the results of another. We aim to demonstrate the use of a targeted thin layer-protected ultra-small gold nanoparticles (Au-NPs) to detect cancer in vivo using multimodal imaging with photoacoustic and computed tomography (CT). The thin layer was produced using a mixed thiol-containing short ligands, including MUA, CVVVT-ol, and HS-(CH2)_11_-PEG_4_-OH. The gold nanoparticle was labeled with a heterobivalent (HB) peptide ligand that targets overexpression of epidermal growth factor receptors (EGFR) and ErbB2, hereafter HB-Au-NPs. A human xenograft model of esophageal cancer was used for imaging. HB-Au-NPs show spherical morphology, a core diameter of 4.47 ± 0.8 nm on transmission electron microscopy, and a hydrodynamic diameter of 6.41 ± 0.73 nm on dynamic light scattering. Uptake of HB-Au-NPs was observed only in cancer cells that overexpressed EGFR and ErbB2 using photoacoustic microscopy. Photoacoustic images of tumors in vivo showed peak HB-Au-NPs uptake at 8 h post-injection with systemic clearance by ~48 h. Whole-body images using CT validated specific tumor uptake of HB-Au-NPs in vivo. HB-Au-NPs showed good stability and biocompatibility with fast clearance and contrast-enhancing capability for both photoacoustic and CT imaging. A targeted thin layer-protected gold nanoprobe represents a new platform for molecular imaging and shows promise for early detection and staging of cancer.

## 1. Introduction

Esophageal adenocarcinoma (EAC) is an aggressive disease with a poor 5-year survival rate of between 15–25% [1]. This disease is associated with high morbidity and mortality, thus accurate staging is important to determine the best therapeutic options for patients. EAC often develops in patients without symptoms, such as acid reflux or dysphagia, and many are not enrolled in an endoscopic surveillance program. Magnetic resonance imaging (MRI), computed tomography (CT), and endoscopic ultrasound are frequently used for cancer staging. These imaging modalities detect grossly visible anatomic abnormalities, such as the presence of a mass, to detect cancer, and are not sensitive to small or subtle lesions. By comparison, molecular imaging methods can be developed to detect cancer by visualizing the functional behavior of tumors based on known cellular and molecular signaling pathways. Epidermal growth factor receptors (EGFR) and ErbB2 are transmembrane tyrosine kinase receptors that stimulate epithelial cell growth, proliferation, and differentiation [2,3]. Overexpression of these targets reflects an increase in biological aggressiveness and a higher risk for progression to cancer [4,5,6]. These cell surface targets can be developed for molecular imaging to improve methods of cancer diagnosis and staging.

Multimodal imaging methods are an emerging approach that uses the physical properties of one modality to validate the results of another. Photoacoustic imaging is a hybrid technology that combines optical excitation with acoustic detection to provide deep tissue penetration with high sensitivity [7]. Light is absorbed by tissue contrast agents and results in thermoelastic expansion that generates acoustic waves. Various types of nanostructures are being developed as contrast agents for photoacoustic imaging [8,9,10]. CT is commonly used for diagnostic imaging and requires ionizing radiation to create cross-sectional images [11,12]. Functional CT is performed with use of contrast agents [13], such as barium suspensions [14] and iodinated small molecules [15]. However, these agents can be limited in use by potential nephrotoxicity, patient hypersensitivity, and short circulation times [16,17]. Therefore, the development of a targeted contrast agent that can be used for multimodal imaging with photoacoustic and CT methods may be synergistic for early detection and monitoring progression of cancer. Here, we aim to demonstrate a targeted nanoprobe specific for EGFR and ErbB2 to detect esophageal cancer using multimodal imaging.

## 2. Results

### 2.1. Preparation and Characterization of HB-Au-NPs

**Figure 1:**
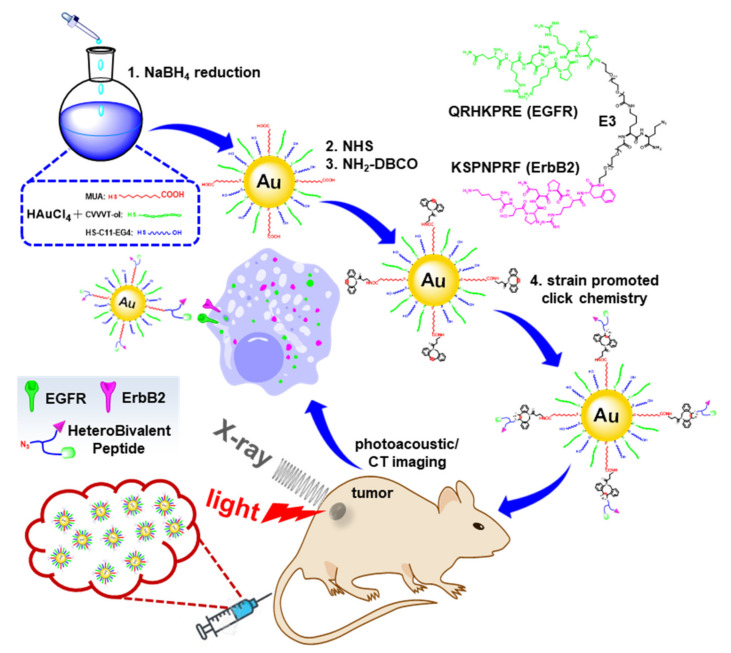
Schematic is shown for labeling the thin layer-protected gold nanoparticles with the heterobivalent (HB) peptide-specific for epidermal growth factor receptors (EGFR) and ErbB2 (HB-Au-NPs) developed for dual photoacoustic/CT imaging.

Thin layer-protected gold nanoparticles (Au-NPs) were synthesized using a one-step facile process, Figure 1. Mixed capping ligands of CVVVT-ol peptide, HS-(CH_2_)_11_-PEG_4_-OH, and 11-mercaptoundecanoic acid (MUA) were self-assembled on the surface of Au-NPs. The terminal carboxylic acid of MUA was activated by an NHS ester and reacted with DBCO-PEG_4_-amine. The azide functionalized heterobivalent (HB) peptide consists of monomers QRHKPRE, hereafter QRH*, and KSPNPRF, hereafter KSP*, specific for EGFR and ErbB2, respectively [18,19,20]. Mass spectra for KSP*-QRH*-E_3_-K-N_3_ is shown, Figure S1A,B. The scheme for synthesis of CVVVT-ol is shown, Figure S1C. The peptides were reacted with alkyne via a strain-promoted azide-alkyne cycloaddition (SPAAC) to modify the surface of the Au-NPs, hereafter HB-Au-NPs.

### 2.2. Nanoparticle Characterization

**Figure 2:**
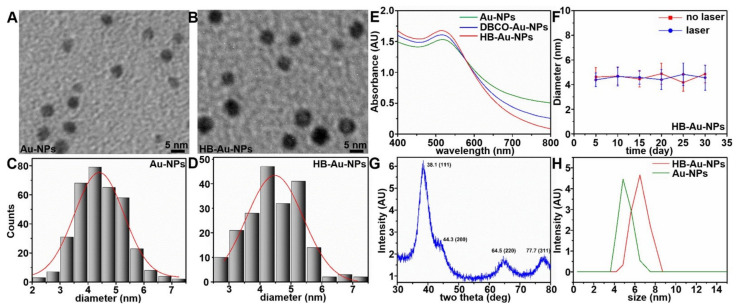
Nanoparticle characterization. TEM images of (**A**) Au-NPs and (**B**) HB-Au-NPs show spherical morphology. (**C, D**) A mean (±SD) diameter of 4.24 ± 0.83 and 4.47 ± 0.88 nm, was measured for Au-NPs and HB-Au-NPs, respectively. (**E**) The absorbance spectra for Au-NPs, DBCO-Au-NPs, and HB-Au-NPs show no shift in the peak at 518 nm. (**F**) No change is seen in the mean (±SD) diameter of HB-Au-NPs in PBS as a function of storage time over 30 days at RT with and without 5 min of laser irradiation (100 µJ/cm^2^). Error bars represent standard deviations of *n* = 3 independent measurements. (**G**) The XRD pattern shows crystalline nanoparticles represented by 4 peaks corresponding to the standard Bragg reflections (111), (200), (220), and (311) of the center faces in a cubic lattice. The peak at 38.1 deg represents preferential growth in the (111) direction. (**H**) Dynamic light scattering (DLS) measurements show mean (±SD) diameter of 6.41 ± 0.73 and 5.50 ± 0.63 nm, respectively, for HB-Au-NPs and Au-NPs.

The nanoparticle properties were characterized using the following methods. Transmission electron microscopy (TEM) showed spherical morphology for Au-NP and HB-Au-NP, Figure 2A,B. The mean diameters of HB-Au-NPs and Au-NPs were 4.24 ± 0.83 and 4.47 ± 0.88 nm, respectively, Figure 2C,D. In the UV absorption spectra, conjugation of the heterobivalent peptide ligand showed negligible change in the peak at 518 nm, Figure 2E. The HB-Au-NPs in PBS solution showed excellent photostability after laser eradiation, Figure 2F, and exhibited good biocompatibility and stability against various endogenous bioactive thiol-containing molecules, Figure S2A–C. An X-ray diffraction (XRD) pattern shows 4 peaks that correspond to standard Bragg reflections from the center faces of a cubic lattice, Figure 2G. The peak at 38.1 deg represents preferential growth in the (111) direction. These results are consistent with a typical purity for crystallinity of the Au nanocrystals. Dynamic light scattering (DLS) showed a mean hydrodynamic size of 5.50 ± 0.63 and 6.41 ± 0.73 nm for Au-NPs and HB-Au-NPs, respectively, Figure 2H, and zeta potential of −12.6 and −9.33 mV, respectively, Figure S3.

### 2.3. In Vitro Cytotoxicity

Cytotoxicity was evaluated by incubating human OE33 (EGFR+/ErbB2+) cancer and Qh-TERT (EGFR-/ErbB2-) benign esophageal cells and SKBr3 (EGFR+/ErbB2+) human breast cancer cells with HB-Au-NPs for 24 h, Figure S4. HB-Au-NPs were found to be non-toxic to cell proliferation at concentrations ranging from 5–400 μg/mL.

### 2.4. Photoacoustic Microscopy of Cells

**Figure 3:**
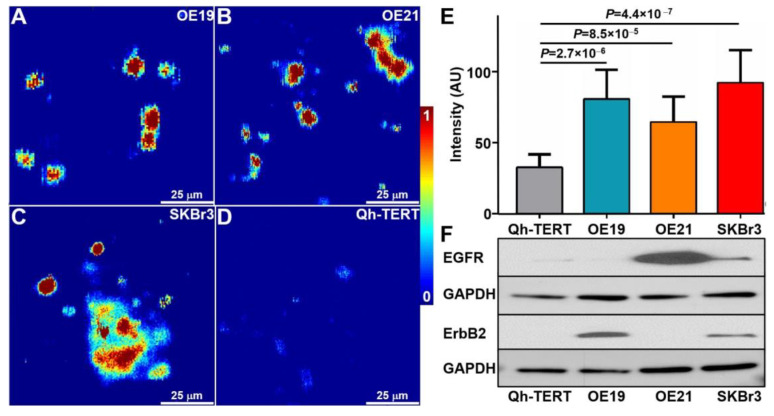
Photoacoustic microscopy. (**A**–**C**) In vitro images of human OE19 (ErbB2+), OE21 (EGFR+), and SKBr3 cells (EGFR+/ErbB2+) incubated with HB-Au-NPs at a concentration of 100 µg/mL show strong signal. (**D**) Image of Qh-TERT cells (EGFR-/ErbB2-) used for control at same concentration shows minimal signal. (**E**) Quantified image intensities are shown. (**F**) Western blot supports EGFR and ErbB2 expression in each cell.

Human OE19 (ErbB2+), OE21 (EGFR+), SKBr3 (EGFR+/ErbB2+), and Qh-TERT (EGFR-/ErbB2-) cells were incubated with 100 µg/mL of HB-Au-NPs. Photoacoustic microscopy images show a strong signal for OE19, OE21, and SKBr3 cells and minimal intensity for Qh-TERT cells, Figure 3A–D. Image intensities for each cell were quantified, and the mean values for OE19, OE21, and SKBr3 were significantly greater than that of Qh-TERT cells. Western blot shows expression levels of EGFR and ErbB2 for each cell type, Figure 3F. Without HB-Au-NPs, we were unable to focus on the cells to capture images.

### 2.5. Photoacoustic Imaging

**Figure 4:**
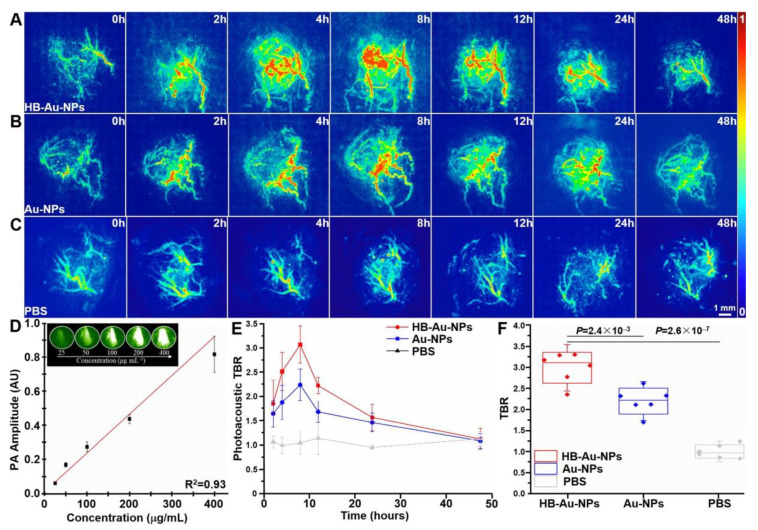
Photoacoustic tomography. In vivo images of human xenograft tumors implanted in mice are shown between 0–48 h post-injection of (**A**) HB-Au-NPs, (**B**) Au-NPs, and (**C**) PBS. (**D**) The in vitro photoacoustic intensity increases linearly with concentration of HB-Au-NPs, *R*^2^ = 0.93. Inset: photoacoustic images are shown of HB-Au-NPs in vials at different concentrations (25, 50, 100, 200, and 400 µg/mL). (**E**) Quantified intensities from the tumors show a peak T/B ratio of 3.08 ± 0.37 and 2.27 ± 0.31 at 8 h post-injection for HB-Au-NPs and Au-NPs, respectively. (**F**) The mean value for HB-Au-NPs is significantly greater than that for either Au-NPs or PBS in *n* = 6 animals, *p* = 2.4 × 10^−3^ and 2.6 × 10^−7^, respectively, by unpaired t-test.

Photoacoustic tomography was performed to evaluate nanoparticle uptake by human OE33 xenograft tumors in vivo following intravenous injection, Figure 4A–C. Images were acquired prior to injection (0 h) to assess background, and a weak signal from intrinsic absorption of oxy- and deoxy-hemoglobin was observed. After systemic injection of HB-Au-NPs, Au-NPs, and PBS, images were collected at 2, 4, 8, 12, 24, and 48 h. The maximum intensity projections (MIP) of the tumors are shown in the coronal view. In vitro photoacoustic intensities measured from HB-Au-NPs in vials demonstrate a linear relationship between photoacoustic signal and HB-Au-NPs concentration up to 400 µg/mL, Figure 4D. In vivo photoacoustic signal of HB-Au-NPs and Au-NPs reached a peak value at 8 h post-injection, Figure 4E. The intensities decreased over time to nearly baseline by 48 h. PBS was injected as control and showed no increase in signal. The mean T/B ratio at 8 h was found to be significantly greater for HB-Au-NPs versus Au-NPs and PBS in *n* = 6 animals, Figure 4F.

### 2.6. CT Imaging

**Figure 5:**
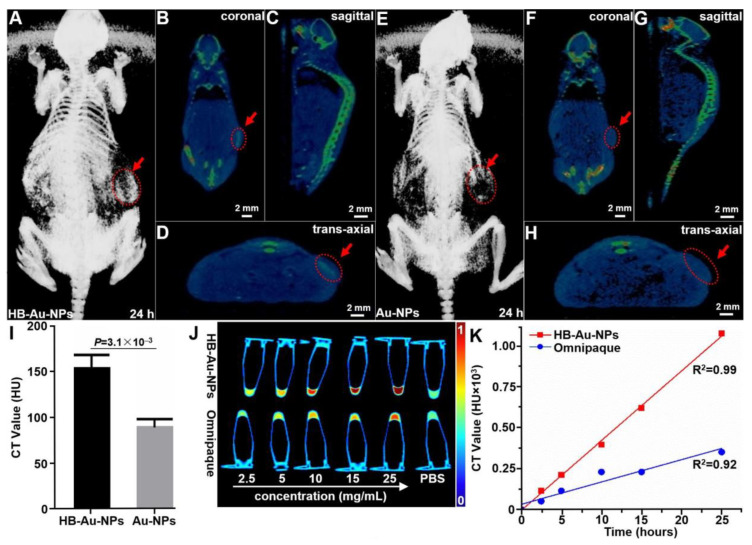
CT images. Representative 3D-reconstructed whole-body CT images at 24 h following intravenous injection of (**A–D**) HB-Au-NPs and (**E–H**) Au-NPs are shown. The locations (arrows) of implanted human OE33 xenograft tumors are shown in the axial, sagittal, and coronal views. (**I**) Quantified results showed a mean value of 155.08 ± 14.94 HU for HB-Au-NPs and 90.47 ± 9.05 HU for Au-NPs from *n* = 3 animals, *p* = 3.1 × 10^−3^ by unpaired t-test. (**J**) CT image of HB-Au-NPs and Omnipaque in vials at different concentrations, including 0 (PBS), 2.5, 5, 10, 15, and 25 mg/mL). (**K**) Intensities increase linearly with concentration of HB-Au-NPs, *R*^2^ = 0.99, and Omnipaque, *R*^2^ = 0.92.

Whole-body CT images were collected to validate nanoparticle uptake by OE33 human xenograft tumors in vivo seen on photoacoustic tomography. At 24 h post-injection, representative images show the tumor (arrows) in different views for HB-Au-NPs, Figure 5A–D, and Au-NPs, Figure 5E–H. The results were quantified, and the mean value from tumor was significantly greater for HB-Au-NPs versus Au-NPs, Figure 5I. The attenuation of CT signal by HB-Au-NPs in vials of deionized water was evaluated and compared with that for Omnipaque, an FDA-approved iodine-based contrast agent used for clinical imaging, Figure 5J. Both contrast agents showed enhanced attenuation with increasing concentration. The intensity for HB-Au-NPs was over 3-fold greater than that for Omnipaque at a concentration of 25 mg/mL, Figure 5K.

### 2.7. Nanoparticle Biodistribution

**Figure 6:**
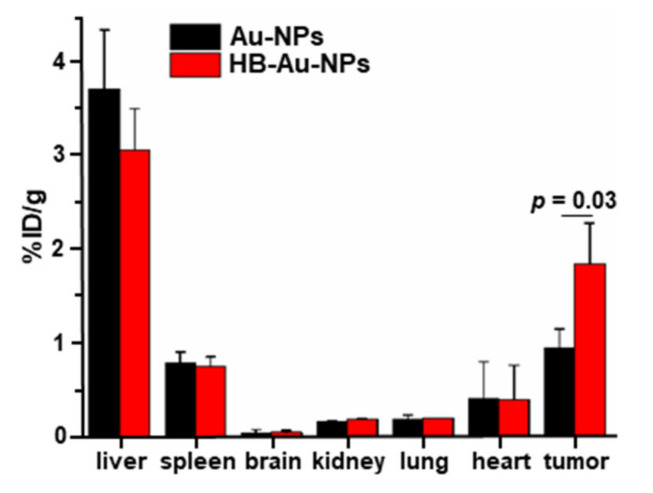
Nanoparticle biodistribution. In vivo uptake of HB-Au-NPs and Au-NPs by major organs, including liver, spleen, brain, kidney, lung, heart, and tumor is shown at 8 h post-injection in mice implanted with human xenograft tumor. The mean value in tumor was significantly higher for HB-Au-NPs than for unlabeled Au-NPs, 1.86 ± 0.43 versus 0.96 ± 0.20 %ID/g, in *n* = 3 animals, *p* = 0.03 by unpaired t-test.

The in vivo biodistribution of HB-Au-NPs and Au-NPs in major organs, including liver, spleen, brain, kidney, lung, heart, and tumor was investigated by inductively coupled plasma mass spectrometry (ICP-MS) at 8 h post-injection on OE33-bearing xenograft nude mice, Figure 6. The mean value for uptake in tumor was significantly greater for HB-Au-NPs versus Au-NPs in *n* = 3 mice. Uptake of both nanoparticles was high in liver and moderate in spleen. These results support nanoparticles being taken up by macrophages and sequestered in the reticuloendothelial system (RES) in part [21].

### 2.8. Animal Toxicity

**Figure 7:**
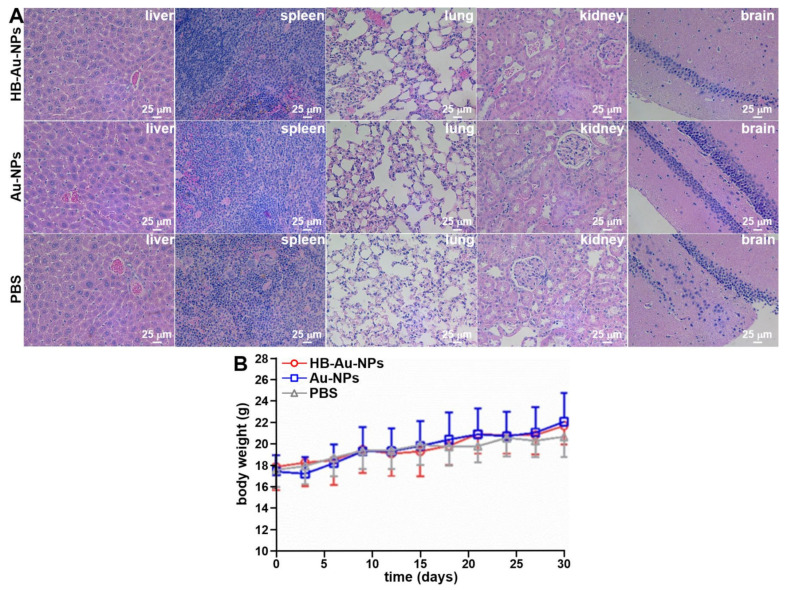
Animal necropsy. Histology (H&E) is shown for major organs, including liver, spleen, lung, kidney, and brain from the mice treated with (**A**) HB-Au-NPs, Au-NPs, and PBS at day 30 following intravenous administration. (**B**) The mean body weight from *n* = 4 animals shows no difference for mice injected with HB-Au-NPs, Au-NPs, and PBS.

Animal necropsy was performed after the completion of imaging, and the main organs, including liver, spleen, lung kidney, and brain, were harvested for necropsy. Histopathology was evaluated to assess toxicity, Figure 7A. No evidence of acute toxicity was observed. Body weight was measured in mice treated with HB-Au-NPs, Au-NPs, and PBS (*n* = 4 animals per group) every 2 days for up to 30 days. The mice gained weight as expected. No significant differences were observed among the different groups, Figure 7B. Laboratory tests, including hematology and chemistry, were evaluated on day 1 and 30 post-injection of HB-Au-NPs and PBS (control) to assess for potential toxicity, Figure S5A,B. No significant difference was seen in any of the results.

## 3. Discussion

Here, we demonstrate use of a targeted gold nanoprobe HB-Au-NPs to detect a human xenograft model of esophageal cancer in vivo using multimodal imaging with photoacoustic and CT. The thin layer-protected gold nanoparticle represents a new platform for biomedical imaging. The thin layer was produced using a mixed thiol-containing short ligands, including MUA, CVVVT-ol and HS-(CH_2_)_11_-PEG_4_-OH. Assembly on the surface of gold nanospheres resulted in greater compactness than conventional larger PEG-coated contrast agents. This feature improves nanoparticle stability under excitation with light. By labeling with a heterodimeric peptide ligand, the gold nanoparticles specifically target overexpression of EGFR and ErbB2 by the cancer cells. The active targeting ability of these nanoparticles is superior to passive tumor uptake via the enhanced permeability and retention (EPR) effect. Peak uptake was observed at 8 h post-injection with systemic clearance by ~48 h. Moreover, these thin layer-protected gold nanoparticles exhibit strong contrast enhancement for both photoacoustic and CT imaging. We found the heterodimer-labeled gold nanoparticle HB-Au-NPs to have excellent stability and biocompatibility. Strong contrast enhancement with both photoacoustic and CT imaging was observed, and the targeting capability of the heterobivalent peptide specific for EGFR and ErbB2 was demonstrated. This nanoprobe may be used as a new platform for diagnosis and staging of cancer. The distribution of EGFR and ErbB2 expression in the xenograft tumor has been previously reported [18]. Peptide monomers are arranged in the heterodimer configuration and are specific for either EGFR or ErbB2 [19].

The thin layer-protected gold nanoparticle with mixed self-assembled monolayers of small ligands represents a novel platform for nanoparticle-based contrast agents [22,23,24]. These compounds have been shown to be extremely stable for biological applications [25,26,27,28]. The thin layer-protected gold nanoparticles were prepared using a simple one-step sodium borohydride reduction without the limitations of a diluted solution. Moreover, the prepared gold nanoparticles can be lyophilized and reconstituted in water without changing characteristic properties. Their unique properties, including small size, large surface-to-volume ratio, tailored surface modification, and excellent biocompatibility, provide utility as a multifunctional biomaterial. As a contrast agent for photoacoustic imaging, gold nanoparticles have higher extinction coefficients than organic dyes at their plasmonic resonance wavelength for greater contrast [29]. Through a thermoelastic expansion mechanism, the absorbed photons can also produce acoustic waves. For CT imaging, the gold nanoparticles resulted in stronger attenuation than Omnipaque, a clinically used iodinated molecule. This effect results from a higher atomic number and electron density (gold: 79 and 19.23 g/cm^3^, respectively, iodine: 53 and 4.9 g/cm^3^, respectively) [30]. Furthermore, gold nanoparticles have a small size and higher CT attenuation. This property may reduce the dosage needed to provide contrast as compared with conventional iodinated agents [31]. Therefore, gold nanoparticles show potential for use as a dual-modal contrast agent both photoacoustic and CT imaging [32,33].

We demonstrated a tumor-targeting strategy by arranging monomer peptides in a dimer configuration and labeled Au-NPs for multi-modal imaging. This ligand structure was designed to improve binding affinity, sensitivity, and specificity [34]. Increased binding affinity may result from a multi-valent effect [18,19]. Higher sensitivity can occur from simultaneous detection of two unique targets. Greater specificity may arise from the dimer binding to a larger target surface area compared to that for the monomer. These improvements may be useful for detecting early targets that are expressed at low levels [20]. Previously, the RGD peptide was labeled with Au-NPs to target α_v_β_3_ integrins expressed by tumor vasculature [35]. Targeted imaging was performed using CT by taking advantage of the high X-ray attenuation properties of the contrast agent. Additionally, Au-NPs have been used to label monomer peptides, such as conjugated analogs of the peptide bombesin [36]. This gastrin-releasing peptide (GRP) binds specifically to GRP receptors overexpressed in breast, prostate, and lung cancers.

Imaging modalities used to diagnose and stage esophageal cancer include barium esophagograms, endoscopic ultrasonography (EUS), computed tomography (CT), magnetic resonance imaging (MRI), and positron emission tomography (PET) [37]. Each modality has specific capability and strengths as well as limitations in terms of sensitivity and resolution. Use of the targeted gold nanoparticle may result in more accurate cancer staging by visualizing the molecular properties of the tumor. The thin layer-protected gold nanoparticles have been synthesized and demonstrated in vivo using the dual-modality photoacoustic and CT imaging. For CT imaging, the small size of the nanoparticle provides higher CT attenuation than larger-sized particles [38]. For photoacoustic imaging, the gold nanoparticles offer advantages over organic dyes in terms of quantum energy transferring coefficient, easy modification, and stability. Laser excitation at λ_ex_ = 532 nm was used to image cells to achieve the best resolution, while λ_ex_ = 680–950 nm was used to image the tumors to maximize image penetration depth.

## 4. Materials and Methods

### 4.1. Materials

Gold (III) chloride trihydrate, sodium borohydride, and 11-mercaptoundecanoic acid (11-MUA) were obtained from Sigma-Aldrich (Burlington, MA, USA). Alkyl PEG (PEGylated-alkanethiol HS-(CH_2_)_11_-EG_4_-OH) was obtained from Prochimia Surfaces (Gdansk, Poland). All these chemicals were used without any further purification. CVVVT-ol peptide was synthesized with DHP-HM-Resin and purified by RP-HPLC. Deionized water used in all experiments was freshly prepared with Milli-Q water purification system (>18 MU cm). Human SKBr3, OE33, OE19, OE21, and Qh-TERT cell lines were obtained from the American Type Culture Collection (ATCC). Cell culture media was procured from Thermo Scientific, unless specifically stated.

### 4.2. Preparation and Characterization of HB-Au-NPs

The Au-NPs was synthesized by borohydride reduction of HAuCl_4_·3H_2_O in presence of a mixture of ligands which are CVVVT-ol peptide (T-ol – threoninol), alkyl PEG (PEGylated alkanethiol HS-(CH2)_11_-EG_4_-OH, sigma-aldrich), and MUA (11-mercaptoundecanoic acid) according to reported protocols [25,27,29]. The concentration of peptides, alkyl PEG and MUA were used in a molar ratio of 40%:40%:20%. Briefly, 0.05 mmol (19.7 mg) of HAuCl_4_·3H_2_O and 0.02 mmol of the ligand mixture containing 0.008 mmol (4 mg) of CVVVT-ol, 0.008 mmol (3.04 mg) of short alkyl PEG, and 0.004 mmol (0.87 mg) of MUA were dissolved in a mixture of solvents, including methanol (3.0 mL) and acetic acid (0.5 mL) by gentle stirring for 5 min, which results in a yellow solution. A freshly prepared sodium borohydride solution (30 mg of NaBH_4_ in 1.5 mL of ice-cooled deionized water) was added dropwise into the above solution under rapid stirring for 4 h at room temperature (RT). Then, 25 µL of 1% Tween-20 was added and gently stirred overnight. The resulting dispersion of functional gold nanoparticles was transferred into a filtration tube (30 KDa MWCO membrane), purified by centrifugation at 3500 rpm, and washed 4 times with methanol containing 0.005% Tween-20 and twice with PBS containing 0.005% Tween 20. The remaining particles were then redispersed in a minimum volume of PBS containing 0.005% Tween-20 and filtered with a syringe filtration unit (0.2 µm) to remove any solid residues. This filtrate was then stored as stock solution. Further dilution of PBS containing 0.005% Tween-20 was carried out to maintain the Au-NPs with OD = 1, and peptide conjugation was conducted at this concentration. For peptide conjugation, about 10 mL of Au-NPs solution was placed into an Eppendorf tube, and 500 µL of EDC and NHS at a concentration of 500 mM each was added. The solution was mixed and purified by centrifugal filtration after 1 h of reaction time and redispersed again in 10 mL of deionized water. DBCO-PEG_4_-amine (0.004 mM, 0.21 mg) was added to this solution, and stirring was performed for 2 h. Unreacted active sites were quenched by adding 200 µL of 0.01 M glycine solution. After an additional 30 min, the nanoparticle solution was purified by centrifugal filtration and redispersed in 10 mL of deionized water. The solution of HB-E_3_-N_3_ (0.004 mM, 11.03 mg) was added, and the reaction was allowed to take place for 12 h. Centrifugal filtration was performed to remove excess heterobivalent peptide, and the solution was washed twice with deionized water.

### 4.3. Nanoparticle Characterization

The samples were dried on carbon-coated copper grids and imaged with a transmission electron microscope (TEM, JEOL JEM-2100) operating at an accelerated voltage of 200 kV. The dimensions of the nanoparticles (*n* = 200) were measured from the TEM images using ImageJ software. UV-vis absorbance was recorded in the spectral range from 400–800 nm at 5 nm increments using a NanoDrop 2000C spectrophotometer at 20° in 10 mm semi-micro cuvettes (Thermo Fisher Scientific, Waltham, MA, USA). The hydrodynamic diameter and zeta potential of nanoparticles were tested with a Zetasizer Nano ZS instrument (Malvern, UK).

### 4.4. In Vitro Cytotoxicity

Cytotoxicity of HB-Au-NPs was evaluated using a Cell Counting Kit-8 (CCK-8, Dojindo Molecular Technologies, Inc., Rockville, MD, USA) assay. Human Qh-TERT and OE33 esophageal cancer cells and SKBr3 human breast cancer cells were investigated with this colorimetric assay. Briefly, the cells were seeded in 96-well plates at a density of ~5 × 10^3^ cells per well in 100 μL of media and incubated overnight at 37 °C in 5% CO_2_. The media in each well was replaced with 100 μL of fresh media containing various concentrations of HB-Au-NPs. After incubation for 24 and 48 h, the media was aspirated, and the cells were washed with PBS twice. Then, 10 μL of CCK-8 solution was added to each well, and incubated for another 2 h at 37 °C. The absorbance was measured using a microplate spectrophotometer (Molecular Devices Tunable Microplate Reader VersaMax, SN# BNR06880) at 450 nm. All concentrations were tested in triplicate. The values were normalized and expressed as percent viability.

### 4.5. Photoacoustic Microscopy of Cells

All cells were maintained at 37 °C and 5% CO_2_ and were supplemented with 10% FBS and 1% penicillin/streptomycin. Human SKBr3 breast cancer cells were cultured in McCoy’s 5A media. Human OE33, OE19, and OE21 esophageal cells were cultured in Roswell Park Memorial Institute (RPMI) 1640 media, and Qh-TERT cells were cultured in keratinocyte-SFM media (Gibco). For photoacoustic microscopy, the cells were fixed in the 4% paraformaldehyde in the bottom of a petri dish filled with PBS. The sample was excited by a pulsed laser (OCT-LK3-BB, Thorlabs, Inc., Newton, NJ, USA) at the excitation wavelength of l_ex_ = 532 nm. An ultrasonic transducer with center frequency of 27 MHz (Optosonic Inc., Arcadia, CA, USA) was immersed into water to detect the photoacoustic signals. The image was obtained by mechanically scanning the objective. The data were analyzed using custom MATLAB (Mathworks) software.

### 4.6. Photoacoustic Imaging

All experimental procedures were performed in accordance with relevant guidelines and requirements of the University of Michigan. Mouse imaging studies were conducted with approval of the University of Michigan Committee on the Use and Care of Animals (UCUCA) under project identification code PRO00009130 with date of approval 8/21/2019. Animals were housed per guidelines of the Unit for Laboratory Animal Medicine (ULAM). Female nude mice at 4 weeks of age were injected in the flank with ~5 × 10^7^ OE33 human esophageal cancer cells in 200 µL of PBS. The mice were imaged when tumor dimensions reached ~5 mm.

Images were collected in vivo using a photoacoustic tomography system (Nexus128, Endra). A tunable pulsed laser (7 ns, 20 Hz, 25 mJ;/pulse) provided excitation wavelengths ranging from l_ex_ = 680–950 nm. The photoacoustic signals were acquired by 128 unfocused 3 mm diameter transducers with 5 MHz center frequency arranged in a helical pattern in a hemispherical bowl filled with water. The console provided data acquisition/reconstruction, servo motors for 3D rotation of the bowl, and a temperature monitor for the water bath. The anesthetized animals were placed in a transparent imaging tray located above the transducers.

### 4.7. CT Imaging

CT images were acquired and reconstructed using an IVIS Spectrum CT instrument (PerkinElmer Imaging Systems) at X-ray voltage of 45 KV and anode current of 500 mA. The images were processed using MicroView (ver 2.5.0) software. Suspensions of HB-Au-NPs and Omnipaque, an iodine-based contrast agent that is FDA-approved for clinical use, containing equivalent concentrations of 2.5, 5, 10, 15, 20, and 25 mg/mL were placed into 0.5 mL Eppendorf tubes. CT images were acquired using an IVIS Spectrum CT. The X-ray voltage was set at 45 kV. A circular ROI was drawn on the coronal view of each tube, and the mean attenuation value for an ROI of 3 slices per tube was recorded and normalized to the value of PBS. The attenuation values for each concentration from *n* = 3 samples were averaged.

### 4.8. Animal Toxicity

Healthy nude mice were injected with HB-Au-NPs and Au-NPs at a dose of 2.5 mg/mL and volume of 100 µL in PBS. The animals were euthanized 30 days post-injection, and major organs, including liver, spleen, lung kidney, and brain, were harvested. The organs were immobilized in 4% paraformaldehyde at 4 °C for 24 h, paraffin-embedded, and cut into 10 mm sections for evaluation by routine histology (Hand E). A total of *n* = 4 mice were used in each group.

### 4.9. Statistical Analysis

All statistical analysis was performed using GraphPad Prism, and plots were generated using Origin 8.0 software.

## 5. Conclusions

We demonstrate use of a heterobivalent peptide labeled with a gold nanoparticle to provide strong contrast enhancement with both photoacoustic and CT imaging. The targeting capability of this multimeric peptide specific for EGFR and ErbB2 was shown in human xenograft tumors in vivo. This nanoprobe shows excellent stability and biocompatibility and has the potential to be used as a new platform for diagnosis and staging of cancer with multi-modal imaging.

## 6. Patents

Patents resulting from the work reported in this manuscript include Wang TD, Chen J. Heterodimeric Peptide Reagents and Methods, WO2019222450A1, https://patents.google.com/patent/WO2019222450A1 (21 November 2019).

## Data Availability

Data is contained within the article.

## Supplementary Materials

The following are available online at https://www.mdpi.com/article/10.3390/ph14111075/s1, Figure S1: Mass spectra, Figure S2: Nanoparticle stability, Figure S3: Nanoparticle parameters, Figure S4: Nanoparticle cytotoxicity, Figure S5: No nanoparticle serum toxicity.
