# Supplementary material for: Thin Layer-Protected Gold Nanoparticles for Targeted Multimodal Imaging with Photoacoustic and CT
Source: Pharmaceuticals (Basel). 2021 Oct 25;14(11):1075. doi: 10.3390/ph14111075 (PMC8624483; doi:10.3390/ph14111075)
Supplement: Supplementary file 1 [file pharmaceuticals-14-01075-s001.zip › pharmaceuticals-1390646-supplementary.pdf]

Supplementary Figures

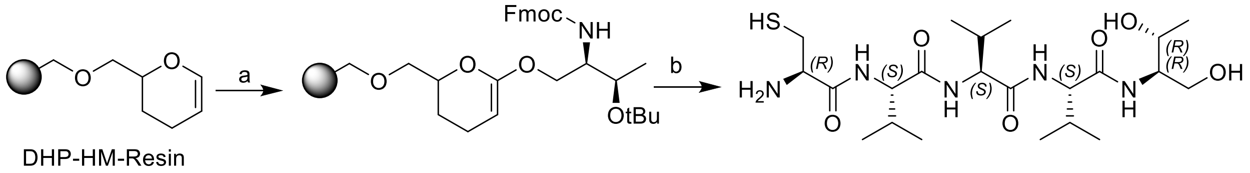

Scheme S1: Synthetic routine of peptide CVVVT-ol. a) PPTS, N_2, 70ºC, DMF, 18h. b) Solid phase peptide synthesis.

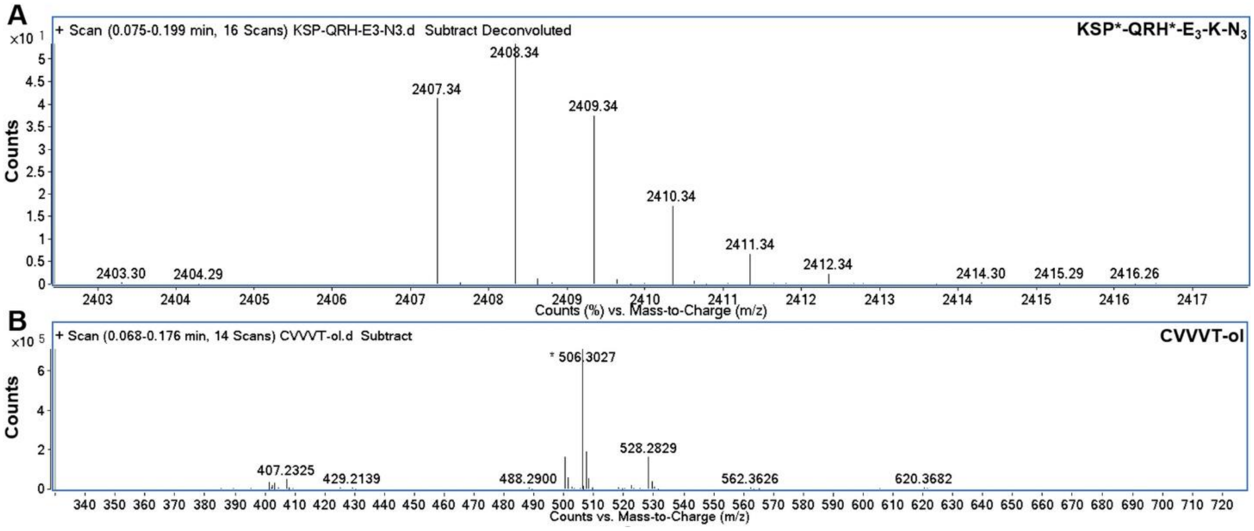

Figure S1 – **Mass spectra**. Results are shown for **A**) KSP*-QRH*-E3-K-N_3, m/z: calculated for $C_{104}H_{174}N_{38}O_{29}$ 2407.33; found 2408.34 [M+1]; and **B**) CVVVT-ol, m/z: calculated for $C_{22}H_{43}N_5OS$ 505.29; found 506.30 [M+1].

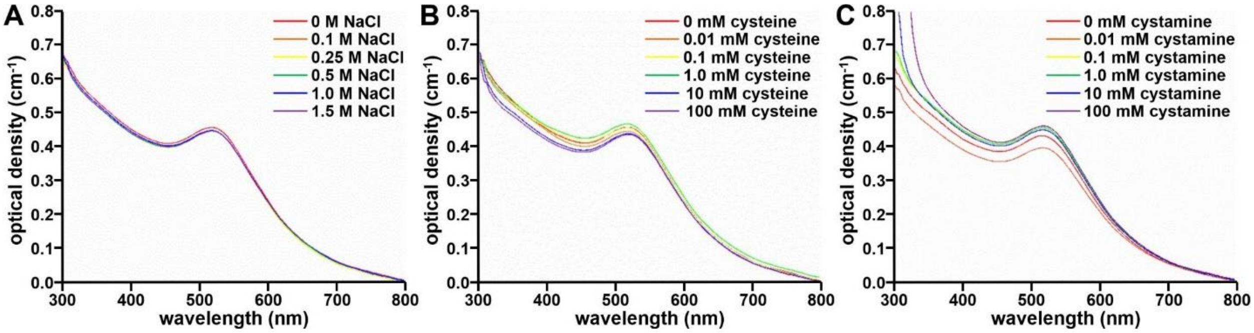

Figure S2 – **Nanoparticle stability**. HB-Au-NPs showed good stability when immersed with endogenous biochemical agents, including **A**) NaCl, **B**) cysteine, and **C**) cystamine over a range of concentrations.

Nanoparticle	Hydrodynamic Diameter (nm)	Zeta Potential (mV)
AuNPs	5.50±0.63	-12.6
HB-AuNPs	6.41±0.73	-9.33

Figure S3 – **Nanoparticle parameters**. DLS measurements of average hydrodynamic diameter and zeta potential of nanoparticles with surface modification in water solution at 25ºC.

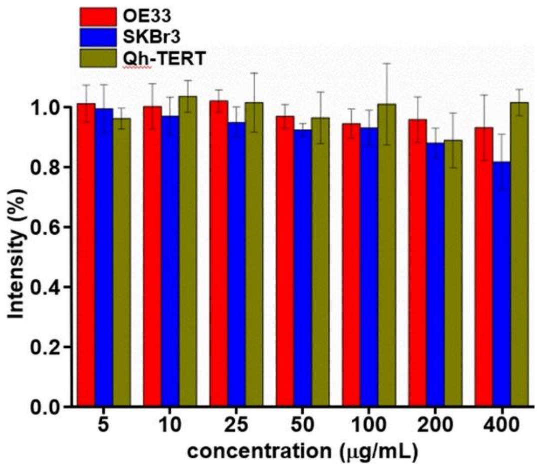

Figure S4 – **Nanoparticle cytotoxicity**. Human OE33 (EGFR+/ErbB2+), SKBR3 (EGFR+/ErbB2+), and Qh-TERT (EGFR-/ErbB2-) cells show no decrease in viability after incubation with up to 400 μg/mL of HB-Au-NPs after 24 hours.

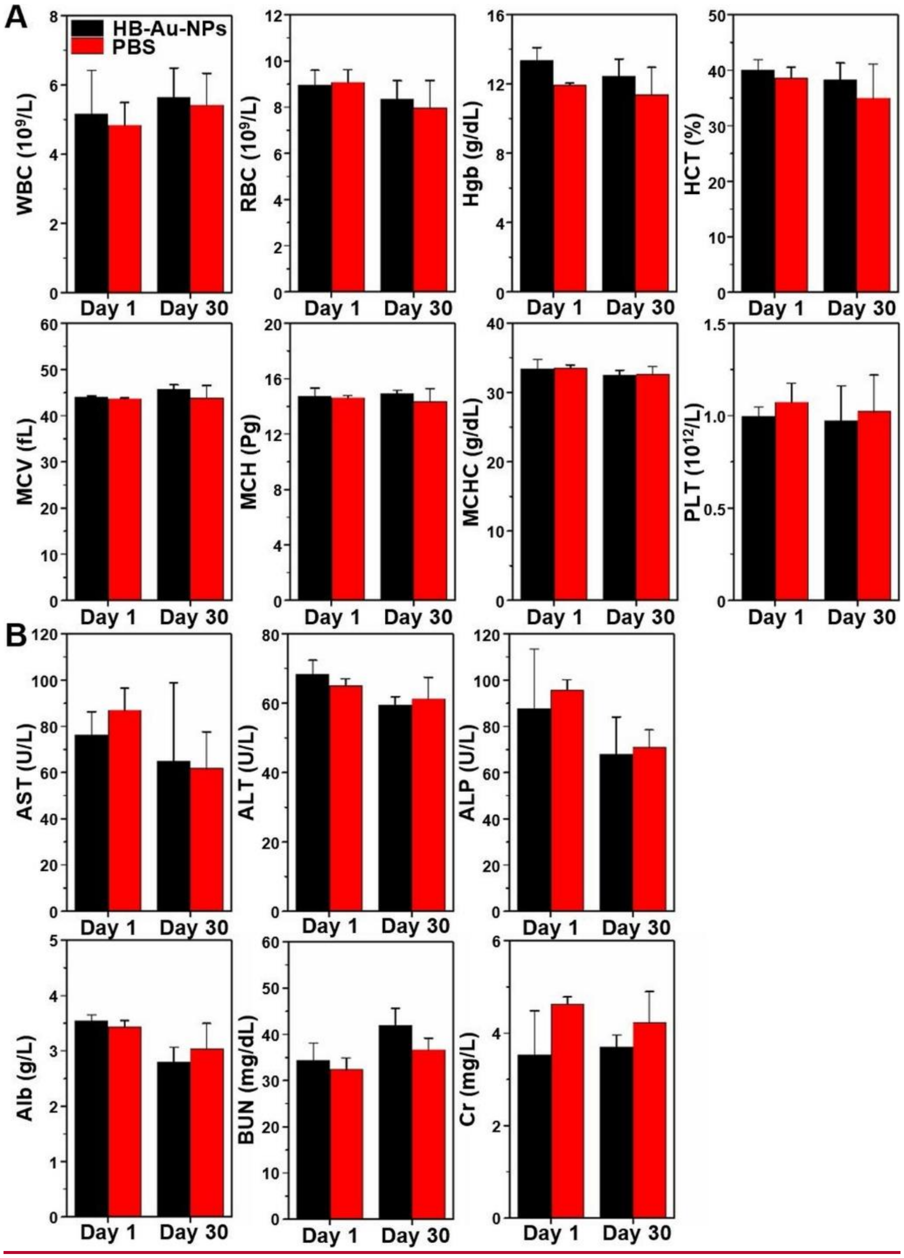

Figure S5 – **Hematology and serum biochemistries**. **A**) Hematology results show no significant changes versus PBS (control) at 48 hours post-injection of HB-Au-NPs, including white blood cells (WBC), red blood cells (RBC), hemoglobin (Hgb), hematocrit (HCT), mean corpuscular volume (MCV), mean corpuscular hemoglobin (MCH), mean corpuscular hemoglobin concentration (MCHC), and platelets (PLT). **B**) Chemistries, including aspartate aminotransferase (AST), alanine aminotransferase (ALT), alkaline phosphatase (ALP), albumin (Alb), blood urea nitrogen (BUN), and creatinine (Cr) also show no significant differences.
